# Risk Factors for Intestinal Invasive Amebiasis in Japan, 2003–2009

**DOI:** 10.3201/eid1805.111275

**Published:** 2012-05

**Authors:** Naoyoshi Nagata, Takuro Shimbo, Junichi Akiyama, Ryo Nakashima, So Nishimura, Tomoyuki Yada, Koji Watanabe, Shinichi Oka, Naomi Uemura

**Affiliations:** National Center for Global Health and Medicine, Tokyo, Japan (N. Nagata, T. Shimbo, J. Akiyama, R. Nakashima, K. Watanabe, S. Oka);; Kohnodai Hospital, Chiba, Japan (S. Nishimura, T. Yada, N. Uemura)

**Keywords:** invasive amebiasis, amebic colitis, parasites, protozoa, prevalence, risk factors, endoscopy, human immunodeficiency virus, CD4 cell count, commercial sex workers, men who have sex with men, Japan

## Abstract

Amebic colitis is increasing among younger men who have syphilis or HIV.

## Medscape CME Activity

Medscape, LLC is pleased to provide online continuing medical education (CME) for this journal article, allowing clinicians the opportunity to earn CME credit. 

This activity has been planned and implemented in accordance with the Essential Areas and policies of the Accreditation Council for Continuing Medical Education through the joint sponsorship of Medscape, LLC and Emerging Infectious Diseases. Medscape, LLC is accredited by the ACCME to provide continuing medical education for physicians.

Medscape, LLC designates this Journal-based CME activity for a maximum of 1 *AMA PRA Category 1 Credit(s)*^TM^. Physicians should claim only the credit commensurate with the extent of their participation in the activity.

All other clinicians completing this activity will be issued a certificate of participation. To participate in this journal CME activity: (1) review the learning objectives and author disclosures; (2) study the education content; (3) take the post-test with a 70% minimum passing score and complete the evaluation at www.medscape.org/journal/eid; (4) view/print certificate.

**Release date: April 11, 2012; Expiration date: April 11, 2013**

## Learning Objectives

Upon completion of this activity, participants will be able to:

- Describe yearly change in prevalence of amebic colitis, based on a Japanese study of persons who underwent endoscopy
- Describe independent risk factors for amebic colitis, based on a Japanese study of persons who underwent endoscopy
- Compare risk factors for amebic colitis between HIV-positive and -negative patients, based on a study of Japanese persons who underwent endoscopy.

## CME Editor

**Thomas J. Gryczan, MS, **Technical Writer/Editor, *Emerging Infectious Diseases. Disclosure: Thomas J. Gryczan, MS, has disclosed no relevant financial relationships*.

## CME Author

**Laurie Barclay, MD, **Freelance writer and reviewer, Medscape, LLC. *Disclosure: Laurie Barclay, MD, has disclosed no relevant financial relationships.*

## Authors

*Disclosures:* ***Naoyoshi Nagata, MD; Takuro Shimbo, MD; Junichi Akiyama, MD; Ryo Nakashima; So Nishimura; Tomoyuki Yada; Koji Watanabe, MD, Ph; Shinichi Oka, MD;*** *and* ***Naomi Uemura, MD,*** *have disclosed no relevant financial relationships.*

## References

[1] Stanley SL Jr. Amoebiasis. Lancet. 2003;361:1025–34. 10.1016/S0140-6736(03)12830-9 12660071

[2] Li E, Stanley SL Jr. Protozoa. Amebiasis. Gastroenterol Clin North Am. 1996;25:471–92. 10.1016/S0889-8553(05)70259-4 8863036

[3] Petri WA Jr, Singh U. Diagnosis and management of amebiasis. Clin Infect Dis. 1999;29:1117–25. 10.1086/313493 10524950

[4] Allason-Jones E, Mindel A, Sargeaunt P, Williams P. *Entamoeba histolytica* as a commensal intestinal parasite in homosexual men. N Engl J Med. 1986;315:353–6. 10.1056/NEJM198608073150603 2874484

[5] Reed SL, Wessel DW, Davis CE. *Entamoeba histolytica* infection and AIDS. Am J Med. 1991;90:269–71. 1996598

[6] Rivera WL, Tachibana H, Kanbara H. Field study on the distribution of *Entamoeba histolytica* and *Entamoeba dispar* in the northern Philippines as detected by the polymerase chain reaction. Am J Trop Med Hyg. 1998;59:916–21. 9886200 10.4269/ajtmh.1998.59.916

[7] Gathiram V, Jackson TF. A longitudinal study of asymptomatic carriers of pathogenic zymodemes of *Entamoeba histolytica.* S Afr Med J. 1987;72:669–72. 2891197

[8] Takeuchi T, Okuzawa E, Nozaki T, Kobayashi S, Mizokami M, Minoshima N, High seropositivity of Japanese homosexual men for amebic infection. J Infect Dis. 1989;159:808. 10.1093/infdis/159.4.808 2538524

[9] Haque R, Huston CD, Hughes M, Houpt E, Petri WA Jr. Amebiasis. N Engl J Med. 2003;348:1565–73. 10.1056/NEJMra022710 12700377

[10] Hung CC, Deng HY, Hsiao WH, Hsieh SM, Hsiao CF, Chen MY, Invasive amebiasis as an emerging parasitic disease in patients with human immunodeficiency virus type 1 infection in Taiwan. Arch Intern Med. 2005;165:409–15. 10.1001/archinte.165.4.409 15738369

[11] Tsai JJ, Sun HY, Ke LY, Tsai KS, Chang SY, Hsieh SM, Higher seroprevalence of *Entamoeba histolytica* infection is associated with human immunodeficiency virus type 1 infection in Taiwan. Am J Trop Med Hyg. 2006;74:1016–9. 16760513

[12] Park WB, Choe PG, Jo JH, Kim SH, Bang JH, Kim HB, Amebic liver abscess in HIV-infected patients, Republic of Korea. Emerg Infect Dis. 2007;13:516–7. 10.3201/eid1303.060894 17552123 PMC2725887

[13] Stark D, van Hal SJ, Matthews G, Harkness J, Marriott D. Invasive amebiasis in men who have sex with men, Australia. Emerg Infect Dis. 2008;14:1141–3. 10.3201/eid1407.080017 18598643 PMC2600324

[14] Ohnishi K, Murata M. Present characteristics of symptomatic amebiasis due to *Entamoeba histolytica* in the east-southeast area of Tokyo. Epidemiol Infect. 1997;119:363–7. 10.1017/S0950268897008236 9440441 PMC2809010

[15] Ohnishi K, Kato Y, Imamura A, Fukayama M, Tsunoda T, Sakaue Y, Present characteristics of symptomatic *Entamoeba histolytica* infection in the big cities of Japan. Epidemiol Infect. 2004;132:57–60. 10.1017/S0950268803001389 14979590 PMC2870078

[16] Amebiais in Japan 2003–2006. Infectious Agents Surveillance Report. 2007;28:103–64.

[17] Okamoto M, Kawabe T, Ohata K, Togo G, Hada T, Katamoto T, Amebic colitis in asymptomatic subjects with positive fecal occult blood test results: clinical features different from symptomatic cases. Am J Trop Med Hyg. 2005;73:934–5. 16282306

[18] Yoshikawa I, Murata I, Yano K, Kume K, Otsuki M. Asymptomatic amebic colitis in a homosexual man. Am J Gastroenterol. 1999;94:2306–8. 10.1111/j.1572-0241.1999.01325.x 10445573

[19] Morán P, Ramos F, Ramiro M, Curiel O, Gonzalez E, Valadez A, Infection by human immunodeficiency virus-1 is not a risk factor for amebiasis. Am J Trop Med Hyg. 2005;73:296–300. 16103593

[20] Seeto RK, Rockey DC. Amebic liver abscess: epidemiology, clinical features, and outcome. West J Med. 1999;170:104–9. 10063397 PMC1305450

[21] Mitarai S, Nagai H, Satoh K, Hebisawa A, Shishido H. Amebiasis in Japanese homosexual men with human immunodeficiency virus infection. Intern Med. 2001;40:671–5. 10.2169/internalmedicine.40.671 11506315

[22] Blumencranz H, Kasen L, Romeu J, Waye JD, LeLeiko NS. The role of endoscopy in suspected amebiasis. Am J Gastroenterol. 1983;78:15–8. 6295138

[23] Pai SA. Amebic colitis can mimic tuberculosis and inflammatory bowel disease on endoscopy and biopsy. Int J Surg Pathol. 2009;17:116–21. 10.1177/1066896908316380 18499688

[24] Caballero-Salcedo A, Viveros-Rogel M, Salvatierra B, Tapia-Conyer R, Sepulveda-Amor J, Gutierrez G, Seroepidemiology of amebiasis in Mexico. Am J Trop Med Hyg. 1994;50:412–9. 8166347 10.4269/ajtmh.1994.50.412

[25] Haque R, Faruque AS, Hahn P, Lyerly DM, Petri WA Jr. *Entamoeba histolytica* and *Entamoeba dispar* infection in children in Bangladesh. J Infect Dis. 1997;175:734–6. 10.1093/infdis/175.3.734 9041357

[26] Nozaki T. Current problems of amebiasis in Japan and recent advances in amebiasis research. Jpn J Infect Dis. 2000;53:229–37. 11227020

[27] Amebic dysentery April 1999–December 2002. Infectious Agents Surveillance Report. 2003;24:79–80.

[28] Takeuchi T, Kobayashi S, Asami K, Yamaguchi N. Correlation of positive syphilis serology with invasive amebiasis in Japan. Am J Trop Med Hyg. 1987;36:321–4. 3826490 10.4269/ajtmh.1987.36.321

[29] Lowther SA, Dworkin MS, Hanson DL. *Entamoeba histolytica*/*Entamoeba dispar* infections in human immunodeficiency virus–infected patients in the United States. Clin Infect Dis. 2000;30:955–9. 10.1086/313811 10880314

[30] HIV/AIDS in Japan in 2009. Infectious Agents Surveillance Report. 2010;31:226–7.

[31] Joint United Nations Program on HIV/AIDS. UNAIDS report on the global AIDS epidemic changes in the incidence rate of HIV infection, 2001 to 2009, selected countries [cited 2011 Apr 15]. http://www.unaids.org/globalreport/Global_report.htm

