# Supplementary material for: Risk Factors for Intestinal Invasive Amebiasis in Japan, 2003–2009
Source: Emerg Infect Dis. 2012 May;18(5):717–24. doi: 10.3201/eid1805.111275 (PMC3358059; doi:10.3201/eid1805.111275)
Supplement: Related texts

# Research article: Risk Factors for Intestinal Invasive Amebiasis in Japan, 2003–2009

**DOI:** 10.3201/eid1805.111275

**Authors:** Naoyoshi Nagata, Takuro Shimbo, Junichi Akiyama, Ryo Nakashima, So Nishimura, Tomoyuki Yada, Koji Watanabe, Shinichi Oka, Naomi Uemura

**Affiliations:** National Center for Global Health and Medicine, Tokyo, Japan (N. Nagata, T. Shimbo, J. Akiyama, R. Nakashima, K. Watanabe, S. Oka);; Kohnodai Hospital, Chiba, Japan (S. Nishimura, T. Yada, N. Uemura)

## Abstract

We determined yearly change in prevalence and risk factors for amebic colitis caused by intestinal invasive amebiasis among persons who underwent endoscopy and assessed differences between HIV-positive and HIV-negative persons in Japan. A total of 10,930 patients were selected for analysis, of whom 54 had amebic colitis. Prevalence was in 2009 (0.88%, 12/1360) compared with 2003 (0.16%, 3/1904). Male sex (odds ratio [OR] 8.39, 95% CI 1.99–35.40), age <50 years (OR 4.73, 95% CI 2.43–9.20), history of syphilis (OR 2.90, 95% CI 1.40–5.99), and HIV infection (OR 15.85, 95% CI 7.93–31.70) were independent risk factors. No differences in risk factors were identified between HIV-positive and HIV-negative patients. Contact with commercial sex workers was a new risk factor among HIV-negative patients. Homosexual intercourse, rather than immunosuppressed status, appears to be a risk factor among HIV-positive patients.

Amebiasis is caused by the protozoan *Entamoeba histolytica*. Each year, this disease develops worldwide in ≈40–50 million persons and causes 40,000 deaths (*1**,**2*). There are several amebic species of protozoans; *E. histolytica* is a pathogenic ameba that can cause invasive intestinal and extraintestinal disease (*1**–**3*). The most frequent manifestations of invasive amebiasis are colitis and liver abscess (*1**,**3**–**5*). Many persons with *E. histolytica* infection are asymptomatic, but invasive disease develops in 4%–10% of persons with symptomatic *E. histolytica* infections over a 1-year period (*1**,**6**–**8*).

Areas with high incidences of amebic infection include India, Africa, Mexico, and parts of Central and South America (*1**,**2**,**9*). In countries with low incidence, such as Taiwan, South Korea, and Australia, invasive amebiasis is uncommon, but reports have indicated that amebiasis is an emerging parasitic infection, particularly among men who have sex with men (MSM) (*10**–**13*). Although epidemics of amebiais have not occurred in Japan, reports from 2001 indicate that invasive amebiasis is common in middle-age men, MSM and HIV-infected patients (*8**,**14**,**15*). In Japan, the prevalence of amebiasis has been increasing according to data from the National Epidemiologic Surveillance of Infectious Diseases (*16*). However, the reasons amebiasis is increasing and the actual prevalence of amebic colitis in daily clinical practice have not been fully clarified. Moreover, some studies in Japan have examined risk factors, but most of these studies have reported case series or case reports without control patients (*14**,**15**,**17**,**18*).

Several studies have indicated that HIV infection is a risk for invasive amebiasis, but no consensus has been reached on this issue (*10**–**12**,**19*). Furthermore, some researchers have suggested that severe invasive amebiasis may develop in HIV-positive patients (*20**–**22*). Susceptibility and clinical factors differ between HIV-positive and -negative patients because of differences in immune status. However, the effect of HIV infection on these risk factors for invasive intestinal amebiasis remains unclear.

To address these issues, we clarified annual changes in prevalence and risk factors for amebic colitis among persons who had undergone endoscopy. These factors were then compared between HIV-positive and HIV-negative patients.

## Methods

### Study Design

We retrospectively reviewed endoscopy records for 14,923 consecutive patients who underwent colonoscopy at the National Center for Global Health and Medicine (NCGM) (Tokyo, Japan) during 2003–2009. Indications for endoscopy included screening for fecal occult blood test; colorectal cancer; anemia; examinations for symptoms such as constipation, loose stool, diarrhea, hematochezia, and abdominal pain; or therapies for colorectal adenoma, early colorectal cancer, and diverticular bleeding.

We excluded patients who had not been tested for HIV infection, syphilis, or hepatitis B virus (HBV) infection. Patients who underwent endoscopic observation only of the anorectal area and those <15 years of age were excluded. A total of 10,930 patients were selected for analysis.

NCGM has 900 beds and is the largest referral center for HIV/AIDS in Japan. Written informed consent for procedures was obtained from all patients before endoscopy and biopsy. The study protocol was approved by the ethics committee of NCGM.

### Sexually Transmitted Diseases

We collected laboratory data for sexually transmitted diseases (STDs), such as HIV infection, syphilis, and HBV infection, before endoscopy. Histories of HBV infection and syphilis were defined as presence of antibody against as hepatitis B surface antigen and positive results in a *Treponema pallidum* hemagglutination test, respectively. In Japan, because only health care workers are vaccinated against hepatitis B, positive results for antibody against hepatitis B surface antigen were attributable to vaccination in few cases.

For HIV-positive patients, we determined CD4 cell counts within 1 week of endoscopy. We categorized CD4 cell counts into 4 groups: >300 cells/μL, 201–300 cells/μL, 101–200 cells/μL, and <100 cells/μL Routes of infection were determined by medical staff who questioned each patient at their first visit to the hospital. Routes were classified into 6 categories: homosexual, bisexual, heterosexual, drug use, untreated blood products, and unknown. We defined sexual preference into 2 categories: MSM and heterosexual. Patients who were not homosexual or bisexual were regarded as heterosexual.

### Diagnosis of Amebic Colitis Caused by *E. histolytic*a Infection

We performed a biopsy and aspirated intestinal fluid from lesions endoscopically when abnormal findings were seen by endoscopy. Amebic colitis was suspected on the basis of endoscopic findings, such as erythema, edematous mucosa, erosions, white exudates, and ulcers (Figure 1) (*22**,**23*). Negative results for intestinal fluid cultures for bacterial species or acid-fast bacillus were confirmed. Amebic colitis was defined as amebic trophozoites in biopsy specimens stained with both hematoxylin and eosin (Figure 2, panel A) and periodic acid–Schiff (Figure 2, panel B), negative intestinal fluid cultures for other species, negative histologic features for other colonic diseases, and a positive clinical response to metronidazole. Trophozoites showed characteristic hemophagocytosis, which is specific for *E. histolytica* infection (Figure 2, panel A).

**Figure 1:** Endoscopic features of amebic colitis, Japan, 2003–2009. A) Colonoscopy showing ulcers in the rectum. B) Colonoscopy showing multiple erosions with exudates surrounded by edematous mucosa in the sigmoid colon.

**Figure 2:** Histologic analysis of amebic colitis, Japan, 2003–2009. A) Trophozoites of *Entamoeba histolytica* ingesting erythrocytes (arrows) (hematoxylin and eosin stain). B) Numerous amebic trophozoites on the mucosal surface (periodic acid–Schiff stain). Original magnification ×200.

### Routes of Amebic Infection

When amebic colitis was diagnosed, the physician asked the patient directly for information about the route of amebic infection. The physician confirmed whether the patient had traveled in tropical areas, resided in a facility for the intellectually disabled, was a male or female commercial sex worker (CSW), or had contact with a CSW or MSM. For travel exposure, history of overseas travel in the past year was elicited. Patients to whom none of the above applied were treated as unknown.

### Statistical Analysis

We assessed changes in annual prevalence by using the χ^2^ test for linear trends. We summarized descriptive data for patients with and without amebic colitis. To determine risk factors for amebic colitis, we estimated the odds ratio (OR) between amebic colitis and clinical factors including age, sex, sexual preference, and history of STDs. We divided patients into 2 age groups, >50 years and <50 years. We used a multiple logistic regression model with factors that showed p<0.2 by univariate analysis. A final model was then developed by backward selection of factors that showed p<0.05. The adequacy of this model was evaluated by using the Hosmer-Lemeshow goodness-of-fit test and a receiver operating characteristic area under the curve.

We also conducted subgroup analysis concerning HIV infection. We investigated interactions between the effect of HIV infection and risk factors for amebic colitis. In HIV-positive patients, the relationship between prevalence of amebic colitis and CD4 cell counts in 4 categories was evaluated by using the χ^2^ test for linear trends. All statistical analyses were performed by using Stata version 10 software (StataCorp LP, College Station, TX, USA).

## Results

### Annual Prevalence of Amebic Colitis

Among 10,930 patients, 54 (0.5%) showed development of amebic colitis. Prevalence was 0.16% in 2003 but tended to increase over time (p<0.01 by trend test) (Figure 3). Prevalence was 5.6-fold higher in 2009 than in 2003.

**Figure 3:** Annual prevalence of amebic colitis, Japan, 2003–2009. Values above bars are no. positive/no. tested.

### Patient Characteristics

HIV-infected patients constituted 248 (2.3%) of 10,930 patients, and they had a median age of 43 years (interquartile range [IQR] 35–55 years) (Table 1). These HIV-infected patients were predominantly male (91.5%, 227/248). Median CD4 cell count was 230 cells/μL (IQR 89.5–401 cells/μL). Routes of HIV infection included homosexual (58.9%, 146/248), heterosexual (12.5%, 31/248), bisexual (10.5%, 26/248), unknown (12.1%, 30/248), untreated blood products (6.0%, 15/248), and drug use (0%).

**Table 1:** Characteristics and risk factors for 10,930 patients with amebic colitis, Japan, 2003–2009*

Characteristic	All, n = 10,930	Amebic colitis, n = 54	No amebic colitis, n = 10,876	Odds ratio (95% CI)
Median age (IQR)	64 (54–73)	41 (36–52)	65 (54–73)	NA
Age, y				
>50	8,875 (81.2)	15 (27.7)	8,860 (81.5)	Referent
<50	2,055 (18.8)	39 (72.2)	2,016 (18.5)	11.4 (6.1–22.4)
Sex				
F	4,522 (41.4)	2 (3.7)	4,520 (39.1)	Referent
M	6,408 (58.6)	52 (96.3)	6,356 (58.4)	18.5 (4.9–156.7)
HIV infection				
Negative	10,682 (97.7)	23 (42.5)	10,659 (98.0)	Referent
Positive	248 (2.3)	31 (57.4)	217 (2.0)	66.2 (36.6–120.7)
HBV infection				
Negative	10,746 (98.3)	47 (87.0)	10,699 (84.0)	Referent
Positive	184 (1.7)	7 (13.0)	177 (1.6)	9.0 (3.4–20.4)
Syphilis				
Negative	10,664 (97.6)	37 (68.5)	10,627 (97.7)	Referent
Positive	266 (2.4)	17 (31.5)	249 (2.3)	19.6 (10.2–36.2)

*Values are no. (%) except as indicated. IQR, interquartile range; NA, not applicable; HBV, hepatitis B virus. p values for all comparisons were <0.05, by Mann-Whitney U test.

Patients with a history of HBV infection constituted 184 (1.7%) of 10,390 patients, and they had a median age of 61 years (IQR 47.5–69 years). These patients were also predominantly male (69.0%, 127/184).

Patients with a history of syphilis constituted 266 (2.4%) of 10,390 patients, and they had a median age of 64 years (IQR 48–74 years). These patients were also predominantly male (76.3%, 203/266).

### Risk Factors for Amebic Colitis

Risk factors for amebic colitis were age <50 years (OR 11.4, 95% CI 6.1–22.4), male sex (OR 18.5, 95% CI 4.9–156.7), HIV infection (OR 66.2, 95% CI 36.6–120.7), history of HBV infection (OR 9.0, 95% CI 3.4–20.4) and history of syphilis (OR 19.6, 95% CI 10.2–36.2) (Table 1). Multivariate analysis showed that age <50 years (OR 4.73, 95% CI 2.43–9.20, p<0.001), male sex (OR 8.39, 95% CI 1.99–35.40, p<0.01), HIV infection (OR 15.85, 95% CI 7.93–31.70, p<0.01), and history of syphilis (OR 2.90, 95% CI 1.40–5.99, p<0.01) were independent risk factors for amebic colitis. This logistic regression model was evaluated by using the Hosmer-Lemeshow test (p = 0.44) and receiver operating characteristic area under the curve (0.90).

### Comparison of HIV-Positive and HIV-Negative Patients

#### Annual Prevalence of Amebic Colitis

Numbers of HIV-positive and HIV-negative patients have been increased annually during 2003–2009 in Japan (Figure 4). Among HIV-positive patients, the prevalence in 2009 increased by 2.1-fold over that in 2003 (Figure 4, panel A). Among HIV-negative patients, the prevalence in 2009 increased by 7.1-fold over that in 2003 (Figure 4, panel B).

**Figure 4:** Annual prevalence of amebic colitis in persons with or without HIV infection, Japan, 2003–2009. A) HIV-positive patients. B) HIV-negative patients. Values above bars are no. positive/no. tested.

#### Risk Factors for Amebic Colitis

Among HIV-positive patients, age <50 years, history of syphilis, and MSM status were risk factors for amebic colitis (Table 2). Immunosuppressed status, such as CD4 cell count <100 cells/μL, was not associated with amebic colitis among HIV-positive patients (Table 2). As CD4 cell counts decreased, the prevalence of amebic decreased (OR 0.3; p = 0.08 by trend test).

**Table 2:** Risk factors for amebic colitis among HIV-positive and -negative patients, Japan, 2003–2009*

Risk factor	HIV-positive patients					HIV-negative patients					p value for interaction
	Amebic colitis, n = 31	No amebic colitis, n = 217	OR (95% CI)	p value		Amebic colitis, n = 23	No amebic colitis, n = 10,659	OR (95% CI)	p value		
Age, y											
>50	6	83	Referent			9	8,777	Referent			
<50	25	134	2.6 (1.0–8.0)	0.04		14	1,882	7.3 (2.9–19.0)	<0.01		0.11
Sex											
F	0	21	Referent			2	4499	Referent			
M	31	196	4.6† (0.8–∞)	0.11†		21	6,160	7.7 (1.9–67.5)	<0.01		0.56
HBV infection											
Negative	26	189	Referent			21	10,510	Referent			
Positive	5	28	1.3 (0.4–3.8)	0.62		2	149	6.7 (0.8–27.9)	<0.01		0.07
Syphilis											
Negative	16	163	Referent			21	10,464	Referent			
Positive	15	54	2.8 (1.2–6.5)	<0.01		2	195	5.1 (0.6–21.1)	0.01		0.48
Sexual preference											
Heterosexual	3	73	Referent			ND	ND	ND	ND	ND	
MSM	28	144	4.7 (1.4–25.0)	<0.01		ND	ND	ND	ND	ND	
CD4 cell count/μL											
>300	14	82	Referent	ND		ND	ND	ND	ND	ND	
201–300	5	31	0.9 (0.3–2.8)	ND		ND	ND	ND	ND	ND	
100–200	9	39	1.35 (0.5–3.4)	ND		ND	ND	ND	ND	ND	
<100	3	65	0.3 (0.07–1.0)	0.15		ND	ND	ND	ND	ND	

*OR, odds ratio; HBV, hepatitis B virus; ND, no applicable data; MSM, men who have sex with men. †Analysis by using exact logistic regression model because number in cell was 0.

Among HIV-negative patients, age <50 years, male sex, history of HBV infection, and history of syphilis were risk factors for amebic colitis (Table 2). No interactions were apparent between HIV infection and risk factors, such as age, sex, history of syphilis, and history of HBV infection.

#### Route of Amebic Infection

Among HIV-positive patients, all 31 patients with amebic infection were male (Table 3). Of these patients, 28 were MSM and 2 were male CSWs. No patients reported contact with CSWs. The route of infection was unknown for 3 patients.

**Table 3:** Route of amebic infection for 54 persons, Japan, 2003–2009*

Route	HIV positive, no. (%), n = 31	HIV negative, no. (%), n = 23
Travelers from tropical areas	0	0
Residents of facilities for intellectually disabled	0	0
MSM, male CSW	28, 2 (90.3)	7 (30.4)
Female CSW	0	2 (8.7)
Contact with female CSW	0	8 (34.8)
Unknown	3 (9.7)	6 (26.1)

*MSM, men who have sex with men; CSW, commercial sex worker.

Among HIV-negative patients, 2 patients were female and 21 were male. Both female patients were CSWs. Of the 21 male patients, 8 had had sexual contact with a female CSW and 7 patients were MSM (2 bisexual and 5 homosexual). The route of infection was unknown for 6 patients.

## Discussion

Endoscopic examination combined with biopsy sample collection is a valuable method for confirming suspected amebic colitis, which is often misdiagnosed as inflammatory bowel disease or other forms of infectious colitis caused by the similarity of associated gastrointestinal symptoms (e.g., diarrhea, hematochezia, and abdominal pain) (*14**,**22**,**23*). However, only a few studies have included patients who had undergone endoscopy (*17**,**22**,**23*). In the present study, we performed a large number of endoscopic examinations. The prevalence of patients with amebic colitis was 0.5% (54/10,930) in this 7-year study. This prevalence was far lower than results from serum prevalence studies, which have shown prevalence in children of 8.4% in Mexico (*24*) and 4.2% in Bangladesh (*25*). However, the annual prevalence of the disease showed a tendency to increase to nearly 1% in recent years, and we assume the prevalence will continue to increase in the future.

In the past, amebic infection in Japan was reportedly caused by overseas travel to countries where epidemics occurred or where amebic infection was found in residents of facilities for the intellectually disabled (*16**,**26*). However, patients with these characteristics were not observed in this study. Multivariate analysis indicated that risk factors for amebic colitis in this study were male sex, age <50 years, and histories of syphilis and HIV infection.

The reason male sex was a risk factor might be related to specific sexual preference (*8**,**10**–**15*) because 52 male patients with amebic colitis often had contact with MSM (n = 35) or female CSWs (n = 8). In this study, MSM constituted 90% of men (OR 4.7 for patients with HIV infection), which is consistent with results of previous reports (*8**,**10**–**15*). However, HIV-negative male patients included heterosexual patients, and ≈35% of them had had contact with CSWs. We included CSWs as routes of infection for amebiasis because amebiasis among female CSWs has been reported in Japan (*27*). Therefore, new infection routes other than MSM, which has been considered a risk because of a diversity of sexual activities, should be considered.

Consistent with results of past reports (*8**,**14**,**15*), younger age was a risk factor. One possibility is that younger age represents a risk factor because younger persons are more sexually active, although this was not clarified in the present study.

Histories of syphilis or HIV infection have been noted as risk factors in previous case series (*7**,**15**,**28*). The present study included many patients with HIV infection or history of syphilis, which supports the hypothesis that these factors increase the risk for amebic colitis.

Among STDs, HIV infection showed the highest risk ratio, a ≈16-fold increase. HIV infection has been identified as a risk factor for invasive amebiasis in many studies (*10**–**12**,**21*), although many details of this risk remain unclear (*19**,**29*).

We presumed that compromised immune function increased the susceptibility of patients to invasive diseases. However, no relationship was seen between low CD4 cell counts and development of amebic colitis. Under existing conditions, the reason for HIV infection representing a risk factor for amebic colitis is considered the preference for oral–anal sex as a common risk factor for both infectious conditions.

We compared prevalence and risk factors between amebic colitis patients with and without HIV infection. An incidence of 0.1% (4/5,193) has been reported in studies of HIV-negative patients with positive results for occult blood in feces (*17*), and our results were similar. However, annual prevalence increased in 2009 (0.38%, 5/1,316) compared with 2003 (0.05%, 1/1,878), and the rate of increase was higher than that for HIV-positive patients. This result calls for careful attention in hospitals in which patients with HIV infection are not commonly encountered. In terms of risk factors, ORs for age, sex, and history of HBV infection or syphilis in our study did not vary according to HIV infection status.

Some limitations need to be considered in this study. First, Japan has not had epidemics of amebiasis, and data in this study were obtained from a metropolitan area. In addition, our hospital treats the largest number of patients with HIV infection in Japan. Second, selection bias was present because participants were patients who had undergone endoscopic examinations, which are highly likely to be performed for healthy patients. In addition, patients suspected before examination of having amebiasis might have been more likely to be actively included in the study. Third, the number of patients with amebic colitis was small; thus, the statistical power of the study might have been low. Fourth, a retrospective design was used for this investigation. With regard to HBV infection or history of syphilis, judgments had to be made for using results of serologic testing in some cases. In addition, determination of sexual preferences and overseas travel had to be based on the self-reports of patients.

In recent years, infectious diseases caused by *E. histolytica* and HIV have been increasing in Japan (*15**,**16**,**30*). HIV infection is a particularly serious problem because its incidence is consistently increasing in Japan while decreasing in western countries (*30**,**31*).

Numbers of patients with both infectious diseases studied are predicted to increase because little is known about measures to prevent infection in association with a diversity of sexual activities. Amebic infection, in particular, is scarcely recognized as a sexually acquired infection, and improved education is needed to prevent these diseases. In Japan, measures to prevent the spread of HIV and amebic infections are urgently needed.

In conclusion, although this study was conducted at 1 center and involved retrospective analysis of a relatively small number of cases of amebic infection, the results suggest that the number of amebic colitis patients with or without HIV infection is tending to increase in Japan. Younger men with syphilis and HIV infections are at increased risk for amebic colitis. Route of infection differed slightly in that contact with CSWs was more frequent among HIV-negative patients than among HIV-positive patients. Among HIV-positive patients, homosexual intercourse, and not immunosuppressed status, seems to be a risk factor for amebic colitis.

## References

[1] Stanley SL Jr. Amoebiasis. Lancet. 2003;361:1025–34. 10.1016/S0140-6736(03)12830-912660071

[2] Li E, Stanley SL Jr. Protozoa. Amebiasis. Gastroenterol Clin North Am. 1996;25:471–92. 10.1016/S0889-8553(05)70259-48863036

[3] Petri WA Jr, Singh U. Diagnosis and management of amebiasis. Clin Infect Dis. 1999;29:1117–25. 10.1086/31349310524950

[4] Allason-Jones E, Mindel A, Sargeaunt P, Williams P. *Entamoeba histolytica* as a commensal intestinal parasite in homosexual men. N Engl J Med. 1986;315:353–6. 10.1056/NEJM1986080731506032874484

[5] Reed SL, Wessel DW, Davis CE. *Entamoeba histolytica* infection and AIDS. Am J Med. 1991;90:269–71.1996598

[6] Rivera WL, Tachibana H, Kanbara H. Field study on the distribution of *Entamoeba histolytica* and *Entamoeba dispar* in the northern Philippines as detected by the polymerase chain reaction. Am J Trop Med Hyg. 1998;59:916–21.988620010.4269/ajtmh.1998.59.916

[7] Gathiram V, Jackson TF. A longitudinal study of asymptomatic carriers of pathogenic zymodemes of *Entamoeba histolytica.* S Afr Med J. 1987;72:669–72.2891197

[8] Takeuchi T, Okuzawa E, Nozaki T, Kobayashi S, Mizokami M, Minoshima N, High seropositivity of Japanese homosexual men for amebic infection. J Infect Dis. 1989;159:808. 10.1093/infdis/159.4.8082538524

[9] Haque R, Huston CD, Hughes M, Houpt E, Petri WA Jr. Amebiasis. N Engl J Med. 2003;348:1565–73. 10.1056/NEJMra02271012700377

[10] Hung CC, Deng HY, Hsiao WH, Hsieh SM, Hsiao CF, Chen MY, Invasive amebiasis as an emerging parasitic disease in patients with human immunodeficiency virus type 1 infection in Taiwan. Arch Intern Med. 2005;165:409–15. 10.1001/archinte.165.4.40915738369

[11] Tsai JJ, Sun HY, Ke LY, Tsai KS, Chang SY, Hsieh SM, Higher seroprevalence of *Entamoeba histolytica* infection is associated with human immunodeficiency virus type 1 infection in Taiwan. Am J Trop Med Hyg. 2006;74:1016–9.16760513

[12] Park WB, Choe PG, Jo JH, Kim SH, Bang JH, Kim HB, Amebic liver abscess in HIV-infected patients, Republic of Korea. Emerg Infect Dis. 2007;13:516–7. 10.3201/eid1303.06089417552123PMC2725887

[13] Stark D, van Hal SJ, Matthews G, Harkness J, Marriott D. Invasive amebiasis in men who have sex with men, Australia. Emerg Infect Dis. 2008;14:1141–3. 10.3201/eid1407.08001718598643PMC2600324

[14] Ohnishi K, Murata M. Present characteristics of symptomatic amebiasis due to *Entamoeba histolytica* in the east-southeast area of Tokyo. Epidemiol Infect. 1997;119:363–7. 10.1017/S09502688970082369440441PMC2809010

[15] Ohnishi K, Kato Y, Imamura A, Fukayama M, Tsunoda T, Sakaue Y, Present characteristics of symptomatic *Entamoeba histolytica* infection in the big cities of Japan. Epidemiol Infect. 2004;132:57–60. 10.1017/S095026880300138914979590PMC2870078

[16] Amebiais in Japan 2003–2006. Infectious Agents Surveillance Report. 2007;28:103–64.

[17] Okamoto M, Kawabe T, Ohata K, Togo G, Hada T, Katamoto T, Amebic colitis in asymptomatic subjects with positive fecal occult blood test results: clinical features different from symptomatic cases. Am J Trop Med Hyg. 2005;73:934–5.16282306

[18] Yoshikawa I, Murata I, Yano K, Kume K, Otsuki M. Asymptomatic amebic colitis in a homosexual man. Am J Gastroenterol. 1999;94:2306–8. 10.1111/j.1572-0241.1999.01325.x10445573

[19] Morán P, Ramos F, Ramiro M, Curiel O, Gonzalez E, Valadez A, Infection by human immunodeficiency virus-1 is not a risk factor for amebiasis. Am J Trop Med Hyg. 2005;73:296–300.16103593

[20] Seeto RK, Rockey DC. Amebic liver abscess: epidemiology, clinical features, and outcome. West J Med. 1999;170:104–9.10063397PMC1305450

[21] Mitarai S, Nagai H, Satoh K, Hebisawa A, Shishido H. Amebiasis in Japanese homosexual men with human immunodeficiency virus infection. Intern Med. 2001;40:671–5. 10.2169/internalmedicine.40.67111506315

[22] Blumencranz H, Kasen L, Romeu J, Waye JD, LeLeiko NS. The role of endoscopy in suspected amebiasis. Am J Gastroenterol. 1983;78:15–8.6295138

[23] Pai SA. Amebic colitis can mimic tuberculosis and inflammatory bowel disease on endoscopy and biopsy. Int J Surg Pathol. 2009;17:116–21. 10.1177/106689690831638018499688

[24] Caballero-Salcedo A, Viveros-Rogel M, Salvatierra B, Tapia-Conyer R, Sepulveda-Amor J, Gutierrez G, Seroepidemiology of amebiasis in Mexico. Am J Trop Med Hyg. 1994;50:412–9.816634710.4269/ajtmh.1994.50.412

[25] Haque R, Faruque AS, Hahn P, Lyerly DM, Petri WA Jr. *Entamoeba histolytica* and *Entamoeba dispar* infection in children in Bangladesh. J Infect Dis. 1997;175:734–6. 10.1093/infdis/175.3.7349041357

[26] Nozaki T. Current problems of amebiasis in Japan and recent advances in amebiasis research. Jpn J Infect Dis. 2000;53:229–37.11227020

[27] Amebic dysentery April 1999–December 2002. Infectious Agents Surveillance Report. 2003;24:79–80.

[28] Takeuchi T, Kobayashi S, Asami K, Yamaguchi N. Correlation of positive syphilis serology with invasive amebiasis in Japan. Am J Trop Med Hyg. 1987;36:321–4.382649010.4269/ajtmh.1987.36.321

[29] Lowther SA, Dworkin MS, Hanson DL. *Entamoeba histolytica*/*Entamoeba dispar* infections in human immunodeficiency virus–infected patients in the United States. Clin Infect Dis. 2000;30:955–9. 10.1086/31381110880314

[30] HIV/AIDS in Japan in 2009. Infectious Agents Surveillance Report. 2010;31:226–7.

[31] Joint United Nations Program on HIV/AIDS. UNAIDS report on the global AIDS epidemic changes in the incidence rate of HIV infection, 2001 to 2009, selected countries [cited 2011 Apr 15]. http://www.unaids.org/globalreport/Global_report.htm

# Research article: Risk Factors for Intestinal Invasive Amebiasis in Japan, 2003–2009

**DOI:** 10.3201/eid1805.111275

**Authors:** Naoyoshi Nagata, Takuro Shimbo, Junichi Akiyama, Ryo Nakashima, So Nishimura, Tomoyuki Yada, Koji Watanabe, Shinichi Oka, Naomi Uemura

**Affiliations:** National Center for Global Health and Medicine, Tokyo, Japan (N. Nagata, T. Shimbo, J. Akiyama, R. Nakashima, K. Watanabe, S. Oka);; Kohnodai Hospital, Chiba, Japan (S. Nishimura, T. Yada, N. Uemura)

## Medscape CME Quiz

## Earning CME Credit

To obtain credit, you should first read the journal article. After reading the article, you should be able to answer the following, related, multiple-choice questions. To complete the questions (with a minimum 70% passing score) and earn continuing medical education (CME) credit, please go to www.medscape.org/journal/eid. Credit cannot be obtained for tests completed on paper, although you may use the worksheet below to keep a record of your answers. You must be a registered user on Medscape.org. If you are not registered on Medscape.org, please click on the New Users: Free Registration link on the left hand side of the website to register. Only one answer is correct for each question. Once you successfully answer all post-test questions you will be able to view and/or print your certificate. For questions regarding the content of this activity, contact the accredited provider, CME@medscape.net. For technical assistance, contact CME@webmd.net. American Medical Association’s Physician’s Recognition Award (AMA PRA) credits are accepted in the US as evidence of participation in CME activities. For further information on this award, please refer to http://www.ama-assn.org/ama/pub/category/2922.html. The AMA has determined that physicians not licensed in the US who participate in this CME activity are eligible for *AMA PRA Category 1 Credits*™. Through agreements that the AMA has made with agencies in some countries, AMA PRA credit may be acceptable as evidence of participation in CME activities. If you are not licensed in the US, please complete the questions online, print the certificate and present it to your national medical association for review.

## Article Title: Risk Factors for Intestinal Invasive Amebiasis in Japan, 2003–2009

## Medscape CME Questions

**1. Based on the Japanese study by Dr. Nagata and colleagues, which of the following statements about prevalence of amebic colitis is most likely correct?**

A. Overall, there was no significant increase in prevalence during the study period

B. During the study period, 0.5% of patients selected for analysis had developed amebic colitis

C. Prevalence among HIV-negative patients remained stable from 2003 to 2009

D. Among HIV-positive patients, the prevalence in 2009 increased 7-fold compared to 2003

**2. You are a public health official asked to consult regarding an increase in prevalence of amebic colitis in Japan. Based on the study by Dr. Nagata and colleagues, which of the following statements about risk factors for amebic colitis is most likely to appear in your report?**

A. Female sex is an independent risk factor

B. Age over 40 years is an independent risk factor

C. History of syphilitic infection is an independent risk factor

D. In multivariate analysis, HIV infection was associated with twice the risk for amebic colitis

**3. Based on the study by Dr. Nagata and colleagues, which of the following statements about differences in risk factors for amebic colitis between HIV-positive and -negative patients would most likely appear in your report?**

A. The risk factor profile was significantly different between HIV-positive and -negative patients

B. Contact with commercial sex workers (CSWs) was not a risk factor among HIV-negative patients

C. Immunosuppressed status was a significant independent risk factor in HIV-positive patients

D. Homosexual intercourse appeared to be a risk factor in HIV-positive patients

## Activity Evaluation

**Table:** 

1. The activity supported the learning objectives.				
Strongly Disagree				Strongly Agree
1	2	3	4	5
2. The material was organized clearly for learning to occur.				
Strongly Disagree				Strongly Agree
1	2	3	4	5
3. The content learned from this activity will impact my practice.				
Strongly Disagree				Strongly Agree
1	2	3	4	5
4. The activity was presented objectively and free of commercial bias.				
Strongly Disagree				Strongly Agree
1	2	3	4	5
